# Evaluation of Hydrogen Gettering Rates Correlated to Surface Composition and Texture of Nickel-Plated Zircaloy Getters of Different Heat Treatment Procedures

**DOI:** 10.3390/molecules28020762

**Published:** 2023-01-12

**Authors:** Ewa C. E. Rönnebro, Mark Engelhard, Danny Edwards, Katarzyna Grubel, Anthony Guzman, Randall Storms

**Affiliations:** Pacific Northwest National Laboratory, Richland, WA 99352, USA

**Keywords:** hydrogen absorption, zircaloy, gettering, nickel, catalyst, surface chemistry

## Abstract

Coatings of metal specimens are known to have an impact on hydrogen gettering (hydrogen absorption). The coating can have one or more functions, such as enhancing gettering, preventing gettering and/or preventing oxidation of the metal substrate. It is known that contaminants and surface texture can impact hydrogen gettering/absorption performance, but has not previously been thoroughly explored. This study evaluated the role of different post-plating heat treatments of nickel-plated zircaloy-4 getters (NPGs) and the role of the heat treatments on gettering rates, surface composition and texture. Nickel plating is applied to prevent oxidation of the Zircaloy-4 surface and also enhances gettering. The nickel plating must be heat treated before desirable gettering can occur. Our NPG getters with historically known satisfying performance were pre-heat treated in air followed by activation heat treatment in a vacuum at a higher temperature. In this study, we were interested in finding out if both heat treatment steps were necessary to obtain a desirable gettering performance, or if one step could be omitted. XPS analysis showed that if the nickel surface is not heat treated before bonding the nickel to the zirconium in the activation step, there will be carbon contaminants on the surface, which significantly reduces gettering. We studied the texture of Zircaloy-4 using SEM/EBSD to compare NPGs with both heat treatment steps with NPGs that had no post-plating heat treatment to learn if the degree of cold work could be impacted by the heat treatment steps. We did not observe any differences in texture between them. We measured gettering rates of both pretreated and activated NPGs and NPGs that had been activated without first being pre-heat treated. We found that the NPGs without the first post-plating heating step had up to a seven times slower gettering rate and obtained higher plateau pressures due to the contaminated surface. Thus, the pre-heat treatment in air before activation is necessary to avoid slower gettering rates and higher plateau pressures.

## 1. Introduction

Hydrogen in metals has been a research topic for several decades, either to store hydrogen for use as an energy carrier or to prevent hydrogen ingress [1,2,3,4,5,6]. Hydrogen can be stored in a variety of different classes of materials aiming for sufficient gravimetric and volumetric energy density that is required for a certain application, such as on-board vehicles, stationery storage or thermal energy storage [4,5]. Hydrogen ingress is a concern in structural materials, such as stainless-steel pipelines, pressure storage tanks and in nuclear reactors because it can result in embrittlement, which reduces the strength of the material.

Zircaloy is used within the nuclear industry as nuclear fuel cladding in light-water-cooled nuclear reactors [7,8,9]. Zirconium was selected for use in nuclear reactors because of its low thermal neutron capture cross-section. The major use of nuclear-grade zirconium tubing is for nuclear fuel rod cladding and radioactive fuel is contained in the cladding tube. The zircaloy cladding is immersed in coolant, which leads to hydrogen ingress from water which eventually leads to embrittlement.

Zirconium is highly reactive and reacts with oxygen to form a zirconium oxide surface layer at ambient conditions. The oxide layer helps protect Zirconium from corrosion, which is another reason Zircaloy has been selected in nuclear reactors to protect nuclear fuel from corrosion [10,11,12,13].

To enhance corrosion resistance, Zircaloy-2 and Zircaloy-4 have been used for many decades, and compositions are well known and commercially available with 98 wt% Zr and 1.5% Sn, 0.1% Cr. Zr-2 contains 0.15% Fe and Zr-4 contains 0.2% Fe. A key compositional difference is that Zircaloy-2 contains 0.05 wt% Nickel and Zircaloy-4 has a higher Iron content of 0.2%, while Zircaloy-2 contains 0.15% Iron. Couet et al. [14] concluded that the hydrogen pickup mechanism is directly linked to the corrosion mechanism, even though hydrogen pick-up kinetics do not follow oxidation kinetics.

Recently developed zirconium alloys with enhanced corrosion resistance include ZIRLO^MT^ from Westinghouse and M5^MT^ from Framatome (former AREVA). They both contain Niobium, but M5 does not contain Tin and Iron. Zirconium and Zircaloy are commercially manufactured for nuclear service to standard specifications ASTM B351 for bars, rods and wire, and ASTM B353 for seamless and welded tubes [15,16]. Both specifications involve melting, forming, cold work and annealing.

Zirconium has an affinity for hydrogen and is therefore a good getter material at elevated temperatures [17]. Hydrogen pick-up fractions are 15% for Zircaloy-4, 12.5% for ZIRLO^TM^ and 10% for M5 [18]. Zircaloy-2 has a lower hydrogen pick-up rate than Zircaloy-4. It is known that surface poisoning by impurity gasses such as H_2_O, CO, and CO_2_ significantly reduces hydrogen absorption rates via the formation of oxides and carbides, which can act as hydrogen barriers and by blocking the access to hydrogen sites on the surface [19].

To prevent zirconium oxide formation, the Zr metal can be coated for protection. If using Zirconium for gettering, the coating should facilitate and not prevent hydrogen ingress. Coatings are often utilized on the surface of a bulk metal or metal alloy specimen in order to either improve or prevent hydrogen absorption, as discussed by Tang et al. and Kashkarov et al. [20,21]. Previously, Zircaloy-4 substrates were modified with nickel–zirconium (NiZr) intermetallics to tailor oxidation performance for specialized applications [22]. The surface modification approach consisted of plating Zr-4 substrates with Ni and performing a thermal treatment to react Ni and Zr-4, similarly to our present study. Thicker Ni plating required longer heat treatments to completely react with the substrate and resulted in thicker intermetallic coatings. Oxidation capacity increased with increased intermetallic thickness, which also led to increased hydrogen pick-up.

The permeation of hydrogen isotopes through solid materials is accepted to proceed via surface adsorption, dissociation into atoms on the surface, diffusion in bulk, and, if permeating through the material, recombination coupled with desorption [1,2,3,6]. Hydrogen can be absorbed by physisorption on the surface, or chemisorption in the bulk of the substrate, which eventually, due to nucleation of the growth process, will result in the formation of hydride phases. There have been numerous studies reporting the rate limiting step of hydrogen absorption and whether it is due to the surface condition and/or the bulk diffusion correlated to limiting steps, including material phase composition, impurities, trapping, defects and dislocations [1,2,3].

Nickel is a known hydrogen absorption catalyst that does not form hydrides, is relatively oxidation resistant and can protect the bulk material from oxidation. Liu et al. reported on the performance of the nickel layer on hydrogen absorption of a Zr-Al getter [23], concluding that it was very important to remove the oxide passivation layer using ion beam sputtering on the zirconium surface before coating to ensure the best hydrogen absorption rates. They also found it is often necessary to “activate” the surface of a material by heat treatment to obtain the best hydrogen absorption performance. The “activation” heat treatment results in the formation of intermetallic compounds in the transition between the layer and the bulk, which facilitates hydrogen diffusion. Liu et al. in 1985 [19] optimized the activation temperature and time to maximize hydrogen pick-up in the getter. This was also discussed by Ichimura et al. in 1987 [24]. We have not been able to find more recent relevant studies related to the composition of the zirconium getter surface composition and intermetallic coatings, texture and heat treatment procedures and its impact on hydrogen gettering. In the hydrogen storage community, coatings have been applied on the storage bulk material with the aim of lowering hydrogenation temperatures, but this topic is beyond the scope of current study.

In this study, we aimed to obtain further insight into correlating getter surface composition and texture with post-fabrication heat treatment procedures and hydrogen absorption rates. The Zircaloy-4 (Zr-4) tube stock used in getter manufacture likely has a preferred microstructural orientation of the grains, or texture, that is developed during the fabrication steps of tube drawing or pilgering. Such processing steps have, as one outcome, the 10–15% cold work. Heating during the getter pretreatment or activation steps could result in some degree of grain recovery, recrystallization or growth, causing a change in texture correlated with a reduction in the cold work. Reduction in cold work is known to cause reduction in mechanical strength and improvement in ductility [25], but the impact on gettering rate is not known.

We measured the gettering rate of nickel-plated Zr-4 getter (NPGs) tubes, determined surface composition using X-ray Photoelectron Spectroscopy (XPS) and the texture of the Zircaloy-4 hydrogen getter using electron backscatter diffraction (EBSD). Both techniques were used to study the possible changes in the microstructure and surface composition due to use of a thermal pre-treatment and/or the activation treatment, and see if this turn impacted the gettering rate. We aimed to clarify if the nickel electroplating procedure introduced impurities on the surface that could possibly reduce hydrogen gettering and to rule out if the pre-treatment step is necessary to remove impurities before the activation heat treatment step. EBSD was used to investigate the texture that arises from tube fabrication, and to see if either of the two heat treatments altered the degree of cold working and texture. Gettering rate tests were performed to determine if pre-heat treatment was necessary before activation in order to obtain optimized hydrogen gettering.

## 2. Results

### 2.1. Getter Rate Tests

Getter rate tests were performed according to a test matrix, see Table 1. Experimental details can be found in Section 4.1 and Section 4.2. Nickel-plated Zircaloy-4 Getters (NPG) of two post-plating treatments were tested: (c) only final activation treatment of tubes 5 and 6, and (d) pretreated in air and activation treatment of tubes 7 and 8. Four specimens per tube were tested; a total of 16 of 2 inch tubes.

#### 2.1.1. Getter Rate Results of Non-Pretreated but Activated NPG c-Samples

**Figure 1:**
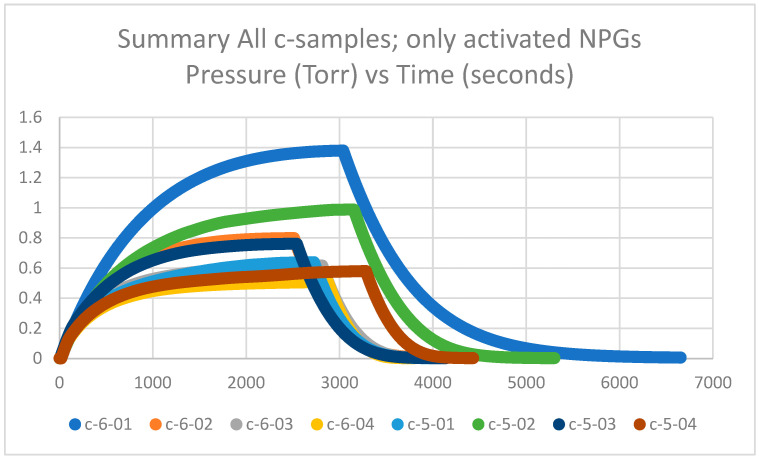
Summary of getter rate tests for all NPG c-samples (getter tubes 5 and 6; only activated) with pressure in Torr vs. time in seconds.

Getter tubes 5 and 6 and labeled c were not pretreated at 425 °C in air but activated at 600 °C in a vacuum. The measured plateau pressures of the eight c-samples are in the range of 0.6–1.4 Torr and are plotted in Figure 1. The plateau pressures are significantly higher than the d-samples by factors of 2 to 6 (see Section 2.1.2). Thus, the calculated getter rates are significantly lower than the d-samples by factors of 2 to 6, as will be explained in Section 2.1.3. It can also be observed that the c-samples have a spread in the data between highest and lowest plateau pressures; however, when compared with historical data of nearly 300 getters in Section 2.1.4, it appears that the spread is within an acceptable range.

#### 2.1.2. Getter Rate Results of Pretreated and Activated NPG d-Samples

**Figure 2:**
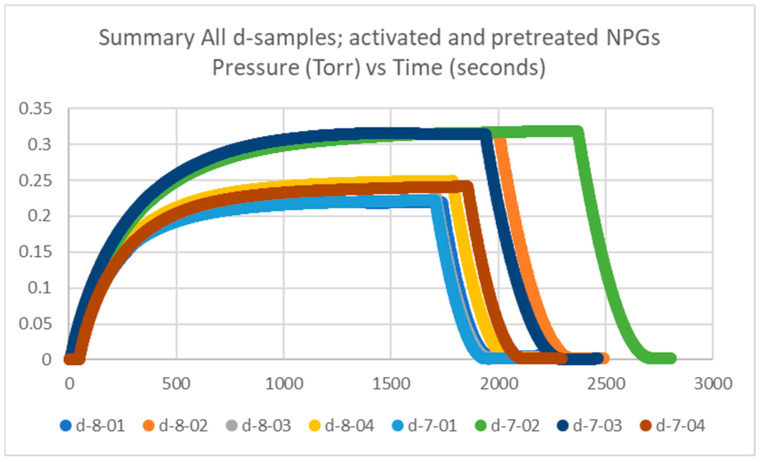
Summary of getter rate tests for all NPG d-samples (getter tubes 7 and 8; pretreated and activated getters) with pressure in Torr vs. time in seconds.

Getter tubes numbered 7 and 8 were both pretreated and activated, and labeled d. The measured plateau pressures of the eight getters are in the range of 0.1–0.3 Torr, up to 0.4 Torr, which is similar to our historical data, which will be discussed in Section 2.1.4. The data for the d-samples is plotted in Figure 2.

#### 2.1.3. Getter Rate Calculations

To better understand the role of the getter pre-treatment step on getter rate (hydrogen absorption rate), we established a simple model using Berkeley Madonna Version 10.2.8 software [26] to fit the measured data to the model, allowing us to compare the getter rates between the samples. Berkeley Madonna is a fast, general purpose differential equations solver. For study purposes, we assumed two equations; splitting of the hydrogen and adsorption of the hydrogen atoms on the metal surface, and we allowed the software to fit the mathematical equation to the obtained data. Constraints in the model were (i) initial pressure of hydrogen gas, (ii) initial concentration of hydrogen atoms, and (iii) timeframe. Values were allowed to freely refine for forward and backward reactions. Since there was no data available on the amount of surface-bound hydrogen atoms, we observed the change in pressure of H_2_ gas.

An assumption was made that only one step is rate-determining, which implies that it is significantly slower than any other step that is involved in the process of hydrogen absorption, as discussed in the Section 1. To compare hydrogen absorption rates between the 16 samples, we chose to study absorption rate after the steady state plateau pressure had been obtained and the initial absorption rate after the hydrogen flow had been shut off to allow the remaining hydrogen in the specimen chamber to be absorbed, limited to a duration of 60 s (see experimental details in Section 4.1 and Section 4.2).

A simple equation of chemisorption was assumed [27] to fit the data to the model.

H_2_ + 2·site → 2H·site

**Figure 3:**
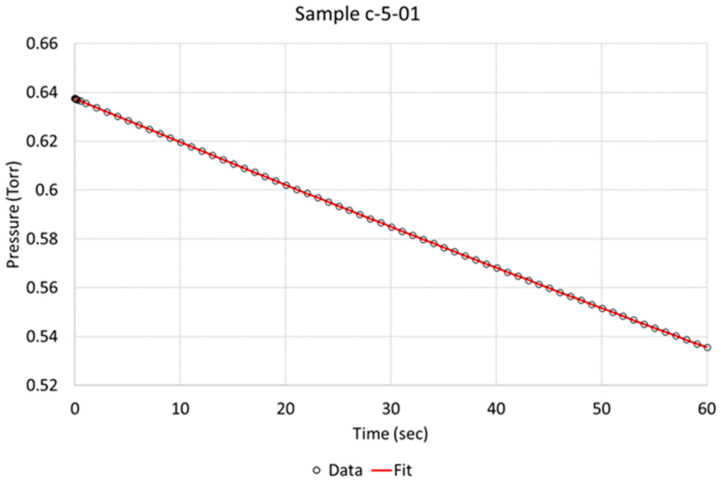
Berkeley Madonna fit of model to observed data indicated by circles for NPG sample c-5-01 (activated).

**Figure 4:**
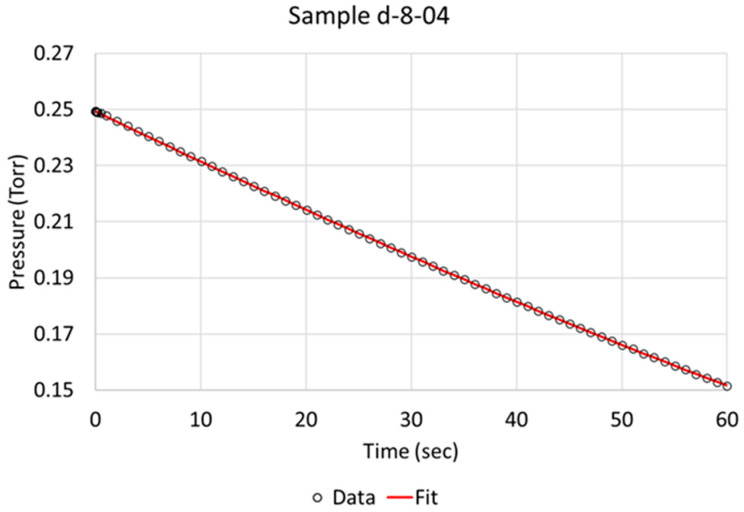
Berkeley Madonna fit of model to observed data indicated by circles for NPG sample d-8-04 (pretreated and activated).

Over the time of interest (60 s), a reasonable fit was obtained, as is exemplified for specimens c-5-01 and d-8-04 in Figure 3 and Figure 4, respectively. Based on the literature and sequence of the hydrogen absorption into metals, it is reasonable to state that the rate-determining step (RDS) is the chemisorption. Therefore, in order to better model the data, another step, i.e., molecular hydrogen being removed due to absorption, had to be added to the model. We define two steps:

Step 1 = K_1_f·(H_2_) − K_1_r·(H^2^)

Step 2 = K_2_f·(2·H)

where H_2_ is molecular hydrogen, H is atomic hydrogen, Kf and Kr are rate constants for the forward and reverse reactions, respectively. Step 1 represents the rate of splitting of H_2_ and recombination of atomic H according to the equation:

H_2_ ⇄ 2H

Step 2 represents the rate of removal of H atom, that we propose to be chemisorption of H on the metal surface.

The calculated hydrogen absorption rates are summarized in Table 2. For clarification, the calculated hydrogen absorption rate is the change of pressure per time; with the assumption of first order kinetics (in which the rate of the reaction depends on the concentration of only one reactant) with the rate constant of the unit of time (s^−^^1^); data was recorded/modeled at one second increments. This simple assessment of the slope during hydrogen absorption of remaining gas allows us to compare rates between samples with different heat treatment (c and d) to be able to correlate the gettering rate with heat treatment procedure and surface composition.

#### 2.1.4. Comparison with Historical Data

**Figure 5:**
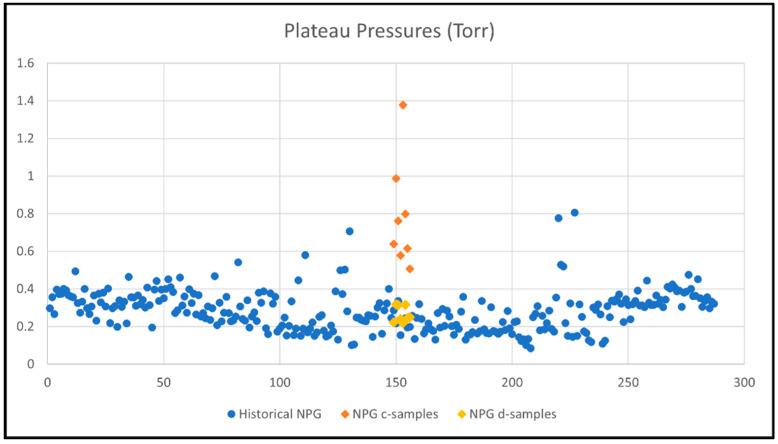
Plateau pressures (y-axis) for historical getter rate tests (280 getters, numbered 1-280 on x-axis) compared with current tests of c-samples (only activated) and d-samples (pretreated and activated).

Historical data of getter hydrogen absorption tests of 2-inch NPG specimens (pretreated and activated similar to d-samples) shows plateau pressures at 0.15–0.4 Torr with a few outliers. The d-samples are within the range of this data. When studying the plots in Figure 5, it is evident that the c-samples depart from the core of the historical data with higher plateau pressures, which is due to not having been pre-treated before activation.

The getter rate tests of the 16 getter specimens show that, initially, only the activated (no pretreatment) c-samples do not pick up hydrogen, as well as both pretreated and activated d-samples and, therefore, the plateau pressures of the c-samples are in the range of 2–6 times higher than the d-samples. The impact of contaminates on the gettering rate will be discussed in Section 2.2. Higher plateau pressures result in gettering rates that are 5–7 times higher for d-samples relative to c-samples. The data has a spread, which can be explained as within the acceptable range when compared with historical data and due to variation in nickel plating thickness.

- Activated-only NPG c-samples have higher plateau pressures, by 2–6 times, than pretreated and activated NPG d-samples;
  o c-samples Range: 0.506–1.379 Torr
  o d-samples Range: 0.241–0.318 Torr
- Activated-only NPG c-samples have lower getter rate constants by 5–7 times than pretreated and activated NPG d-samples;
  o c-samples Range: 1.31·10^−3^–2.89·10^−3^ s^−^^1^
  o d-samples Range: 5.93·10^−3^–8.79·10^−3^ s^−^^1^

### 2.2. XPS-Analysis

Selected areas on each of the samples were examined with X-ray photoelectron spectroscopy (XPS) to characterize the chemical composition of the surface of nickel-plated getter tube samples. The intent of these examinations was to examine the surfaces for evidence of substances that may be detrimental to dissociation and/or diffusion of hydrogen, and whether these substances are modified or removed by the pretreatment and/or thermal activation process. Table 3 shows the test matrix for XPS analysis of the post-plating heat-treated NPG samples.

#### 2.2.1. No Heat Treatment NPG a-Tubes

**Figure 6:**
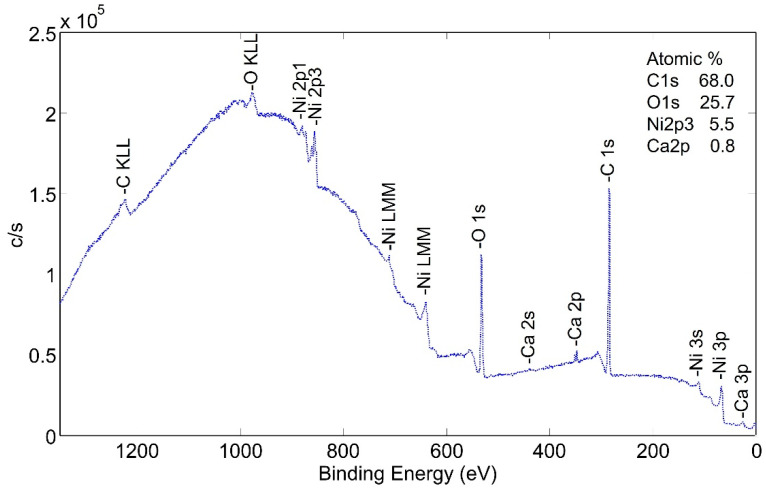
XPS wide scan (low energy) photoemission spectra of non-heat-treated NPG a-sample.

Surface species of an as-plated a-sample without following heat treatment were studied. XPS wide scan photoemission spectra (Figure 6) show the presence of carbon, nickel and oxygen. It is important to note that the peak heights are not proportional to the percentage of each element on the surface, but valid comparisons can be drawn between scans as the condition of the surfaces is modified.

The same sample was sputtered with 2 kV monoatomic Ar+ ion and the spectra dramatically changed as the predominant peaks were those of nickel. The depth of the carbon-rich layer was not measured with the wide scan technique. Narrow scan analyses were performed in order to better examine the carbon-rich layer. Through several scans, the layer averaged approximately 55 at% C, 30 at% O and 13 at% Ni.

**Figure 7:**
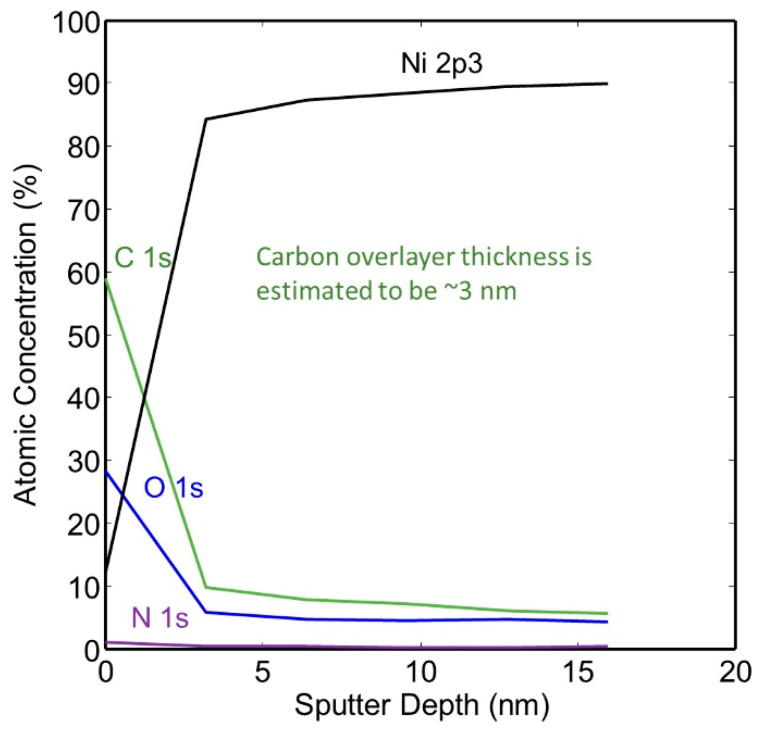
XPS Depth profile of as-plated NPG without heat treatment (a-sample).

The thickness of the hydrocarbon-rich surface layer was calculated from the XPS spectra collected from the surface of the as-plated a-tube and from the compositional XPS depth profiles. Carbon was found to be richest at the surface, and the thickness of the carbon film was calculated to be approximately 3.0 nm. We used the following equation to calculate the overlayer thickness: d = −λC1s, C cosθ ln(1 − x/100) where x = nominal carbon concentration assuming uniform analysis and λ C1s, C = effective attenuation length of C1s photoelectrons in adventitious carbon [28,29,30]. A small amount of carbon was detected on the surface of the nickel plating, but the composition was found to be relatively constant after the first few layers of material were removed. The XPS depth profile of this specimen is given in Figure 7.

#### 2.2.2. Pretreated (Non-Activated) NPG b-Tubes

**Figure 8:**
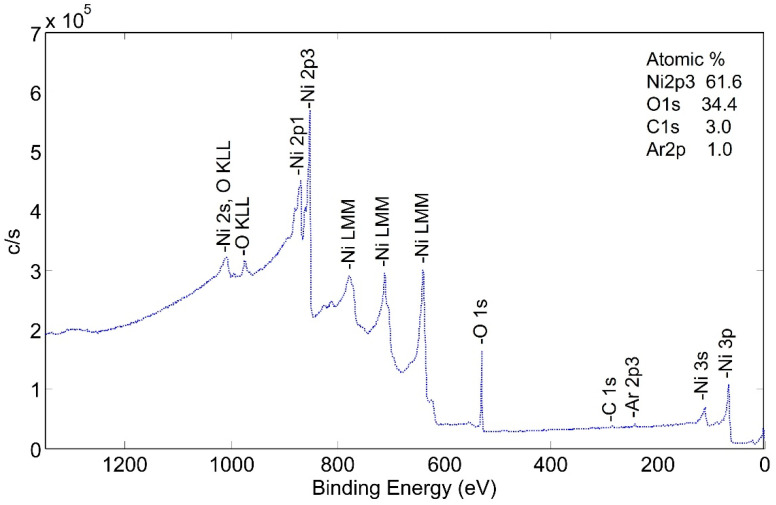
Wide scan XPS spectra for NPG heat treated at 425 °C (b-sample).

This tube section was heated to 425 °C in a flowing mixture of 80% nitrogen and 20% oxygen—simulating air flow. This corresponded to b-tubes that had been pre-heat treated but not activated. The XPS spectra are shown in Figure 8.

Two significant observations can be made from this spectrum: (1) surface carbon was essentially eliminated by the pretreatment process, and (2) the surface appears to be made up of nickel and nickel oxide.

**Figure 9:**
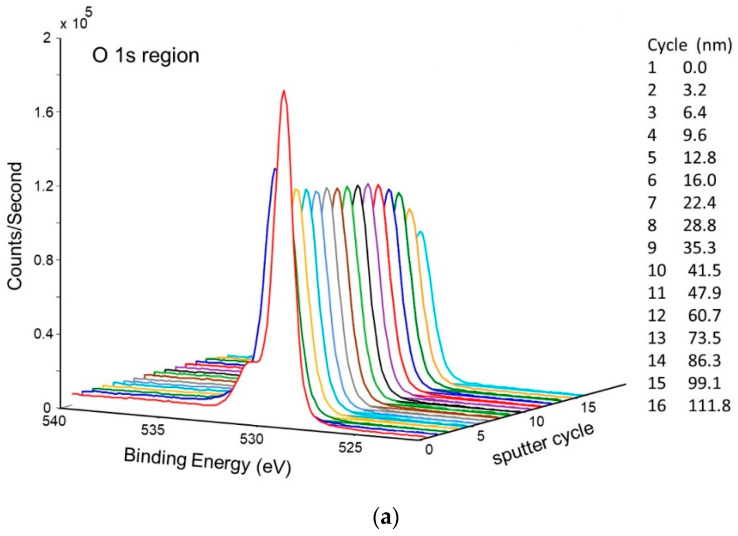

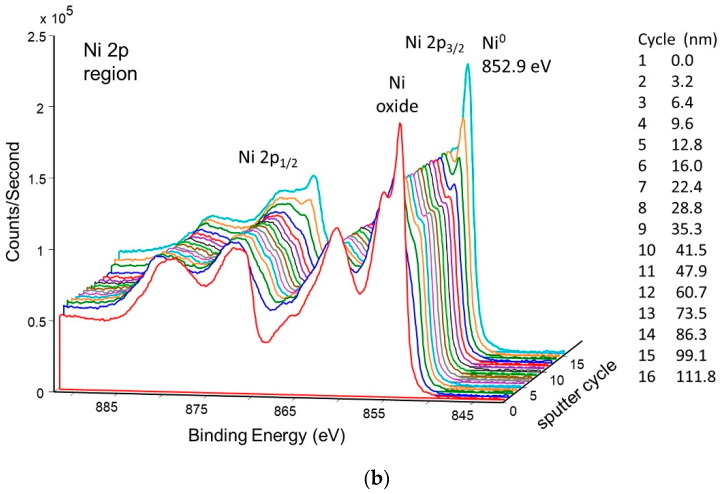
Depth profile of pretreated NPG surface b-sample showing (**a**) corresponding decrease in oxygen, (**b**) change from NiO to Ni.

An XPS sputter profile of the pretreated getter tube was obtained. The results are shown in Figure 9a,b. As seen in Figure 9a, nickel oxide is predominant at the surface, but, after ~10 sputter cycles (just a couple of nm deep), the surface is predominantly metallic nickel. Analysis of oxygen during the sputter profile confirms this conclusion (Figure 9b).

#### 2.2.3. Pretreated and Activated NPG d-Tubes

**Figure 10:**
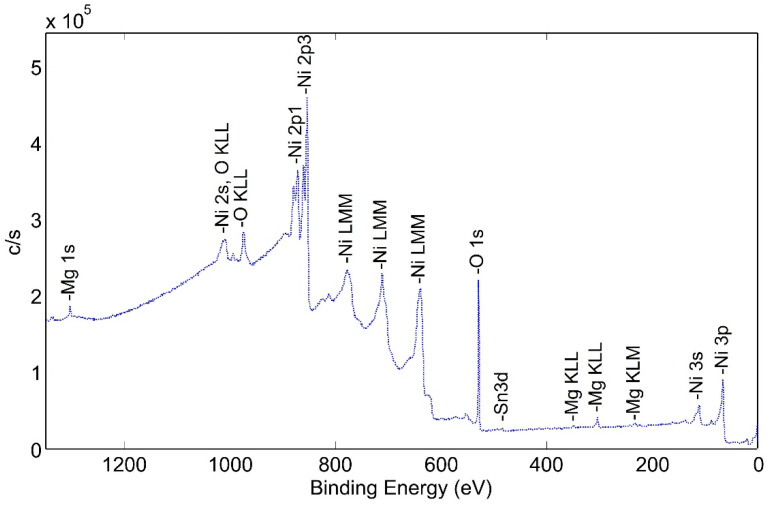
Wide Scan Photoemission Spectra for Surface of Pretreated and Activated NPG d-sample.

This tube section was first heated to 425 °C for 120 min in a flowing mixture of 80% nitrogen and 20% oxygen; then, the atmosphere was pumped down to a high vacuum and the tube section was heated to 600 °C for 60 min. This corresponds to d-tubes with both heat treatment and activation. The XPS spectra of the heat-treated surface are shown in Figure 10.

The height of the oxygen peaks is slightly lower than for the pretreated sample. In addition, small peaks corresponding to magnesium and tin are observed. Although tin may have diffused through the nickel plating from the Zircaloy tube, there is not any indication of zirconium at the outer surface of the tube. The reason for the presence of magnesium is not known but could originate from the electrolytic bath.

**Figure 11:**
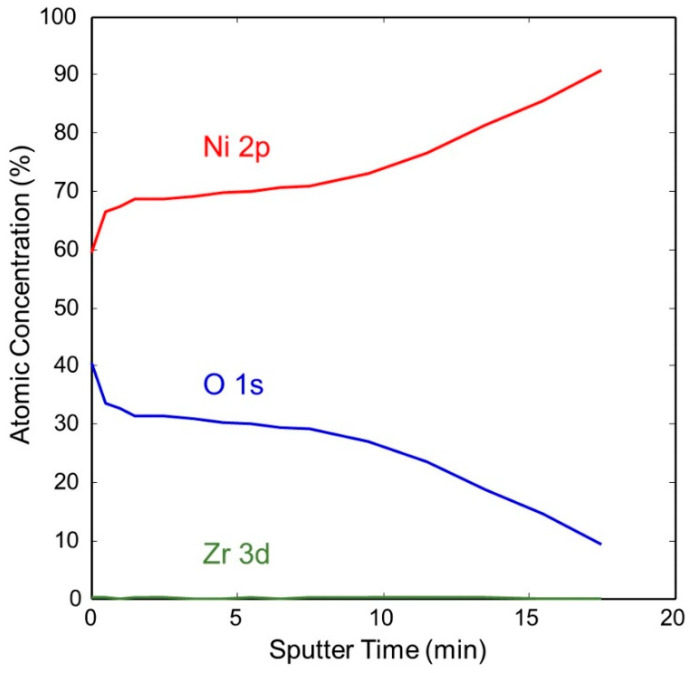
Atomic concentration depth profile of Ni and O for pretreated and activated getter d-sample.

**Figure 12:**
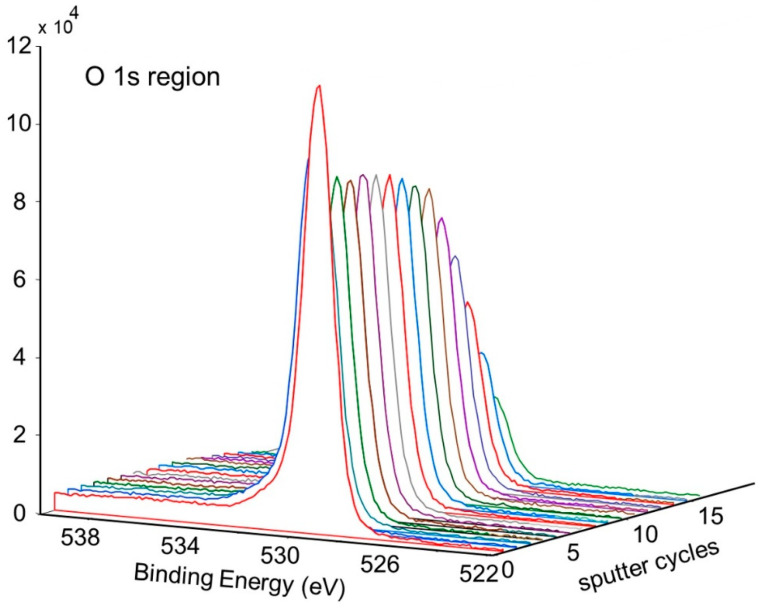
Depth profile for pretreated and activated d-sample showing decrease of NiO and increase of metallic Ni with depth.

**Figure 13:**
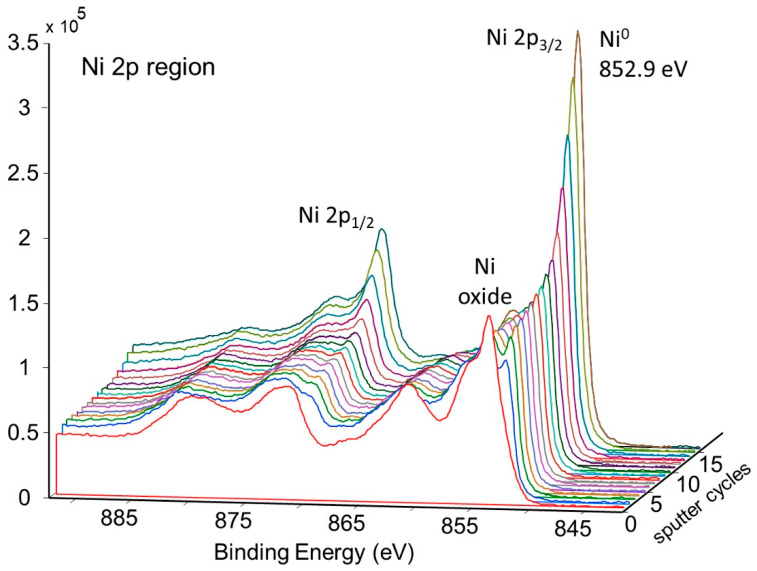
Depth profile for pretreated and activated d-sample showing decrease of NiO with depth.

Figure 11 shows the change in concentration with a depth of Ni and O on the pretreated and activated d-sample. This shows a sharp increase in Ni with a corresponding sharp decrease in O at the surface, followed by a more gradual change with increasing depth. This is also illustrated in Figure 12 and Figure 13, showing the sharp decrease in NiO at the surface with a more gradual increase in metallic Ni with depth.

### 2.3. EBSD Texture Analysis

We performed EBSD texture analysis to learn if heat treatment has an impact on the degree of cold work, which potentially could impact hydrogen gettering rates. To probe the possible effects on the surface of the pre-treatment and activation thermal treatments compared to the as-plated tubes, two samples of each were selected for EBSD. The samples selected are listed below in Table 4.

**Figure 14:**
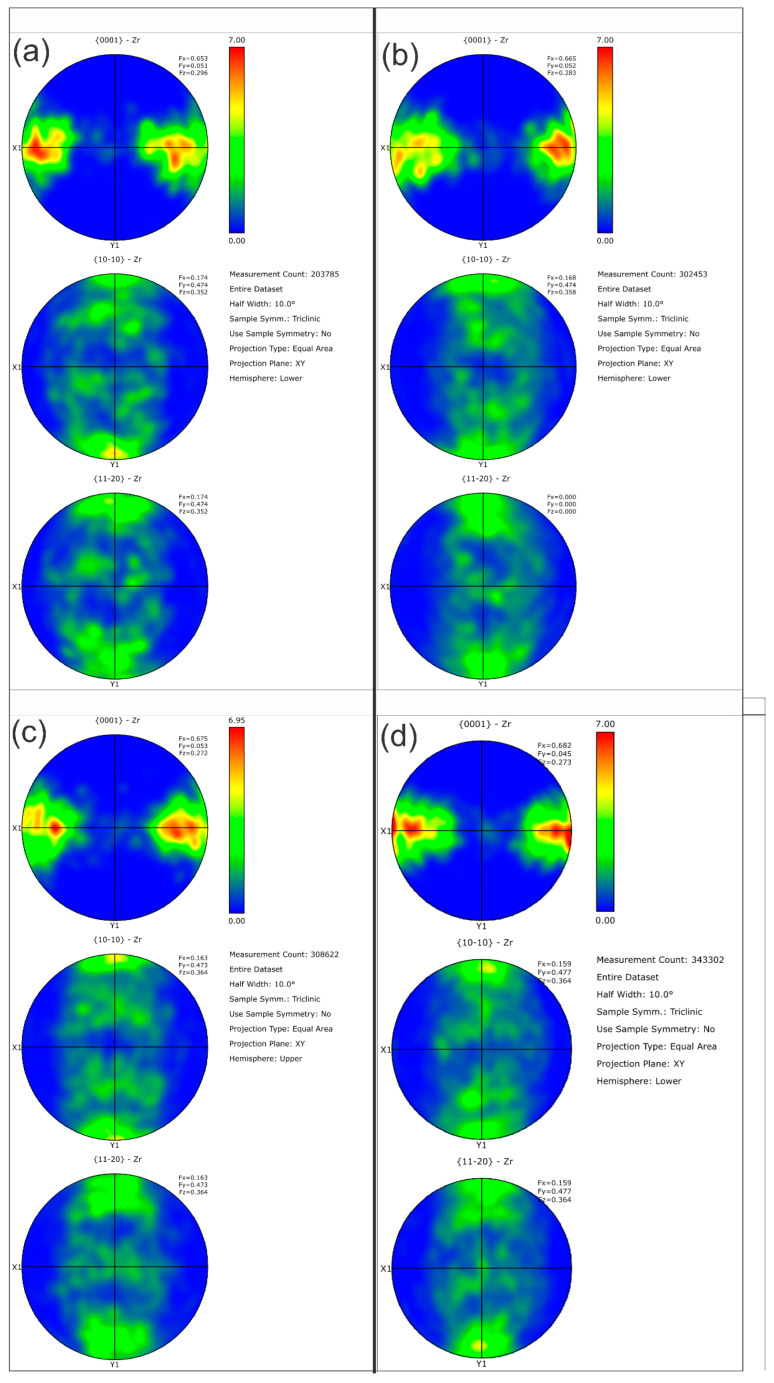
Pole figures are shown for the four NPG tubes: (**a**) Tube a-1-05, (**b**) Tube a-2-05, (**c**) Tube d-7-05 and (**d**) Tube d-8-05. As the Kearn values indicated, there are no discernible differences between the as-plated getter tubes and those given pretreatment and activation.

**Figure 15:**
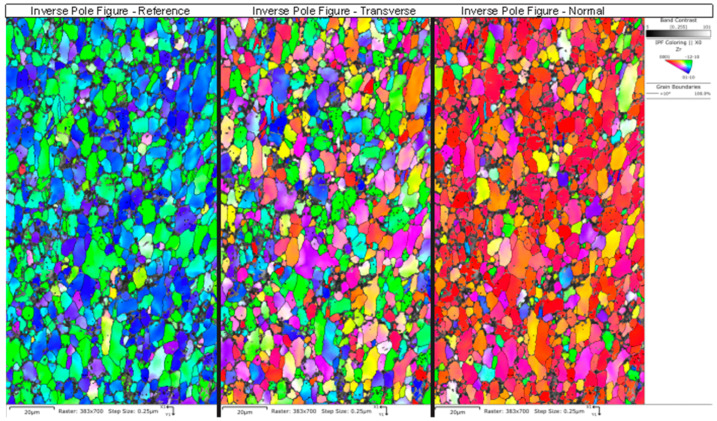
An example is shown of the inverse pole figures maps obtained from NPG sample a-1-05, with the inset color-legend showing the corresponding plane. The IPF map for the normal direction shows mostly red-colored grains due to the basal texture of the drawn tubes. This was consistent across all of the grains.

**Figure 16:**
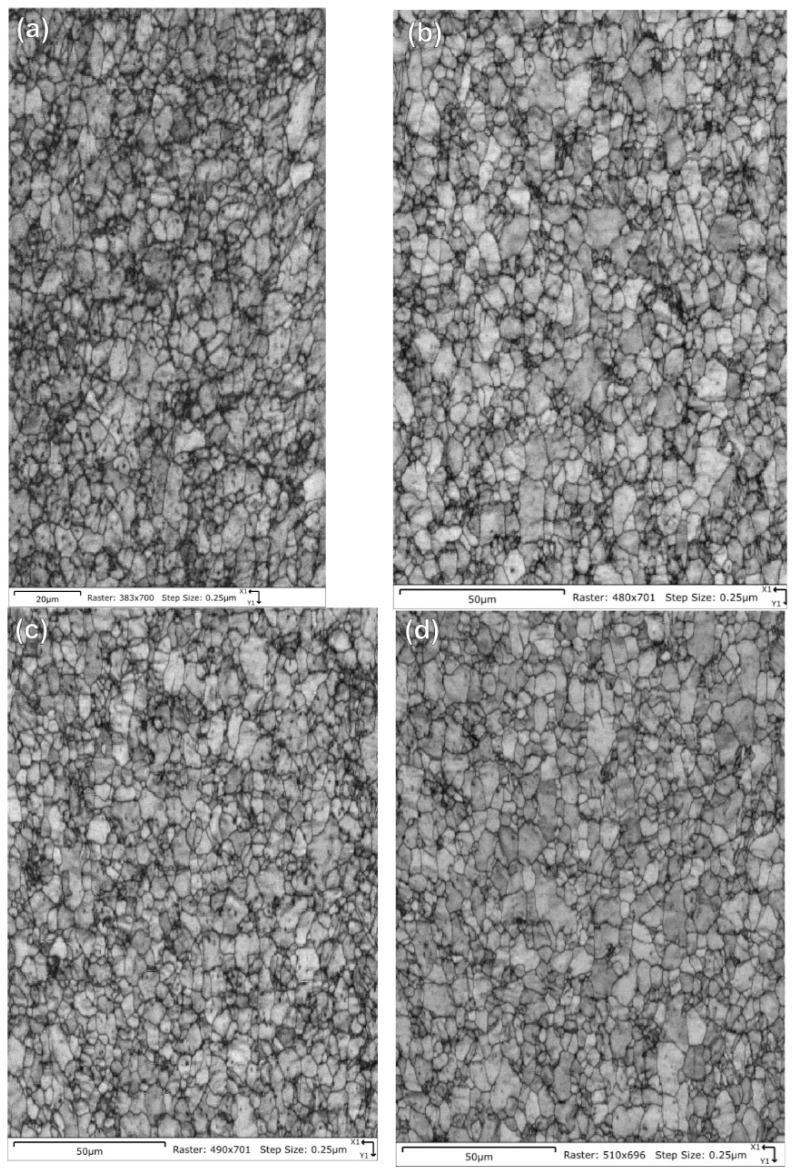
Pattern quality maps are provided for the NPG tubes: (**a**) Tube a-1-05, (**b**) Tube a-2-05, (**c**) Tube d-7-05 and (**d**) Tube d-8-05. The maps do not show significant differences other than a slightly fuzzier grain boundary contrast for tube a-1-05 due to a poorer final surface finish.

**Figure 17:**
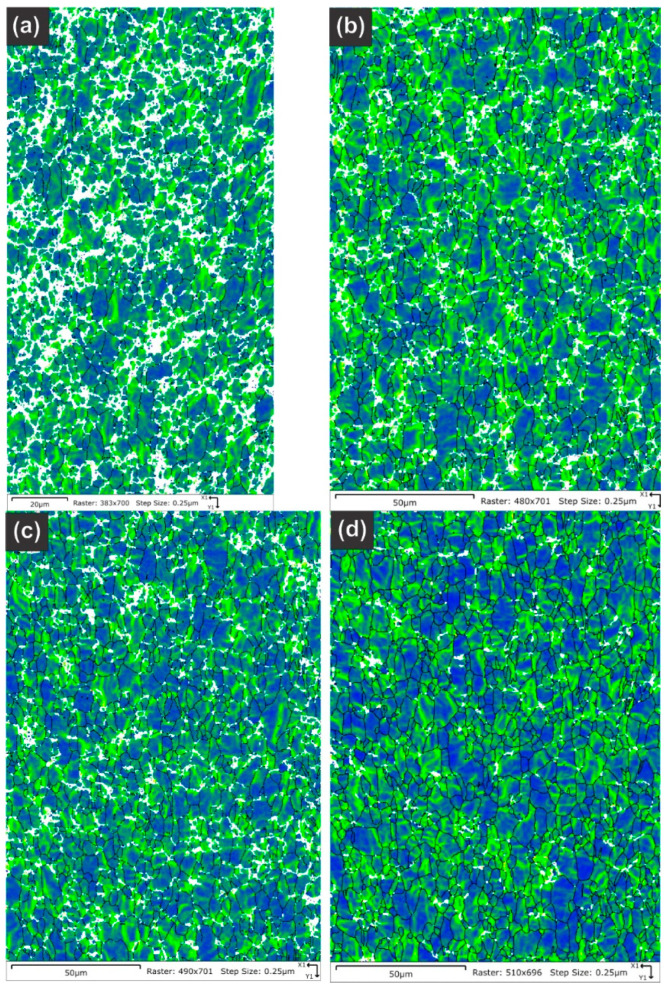
Kernel average misorientation maps are provided for the NPG tubes: (**a**) Tube a-1-05, (**b**) Tube a-2-05, (**c**) Tube d-7-05 and (**d**) Tube d-8-05. Only the sample a-1-05 shows any significant differences, but this is assigned to a poorer final polish rather than any underlying differences in the material as the overall texture and Kearns values were nearly identical between the four samples.

Table 5 summarizes the pole figure measurements and the resulting Kearns values for the four samples in Table 4, as well as the number of grains present in each map and the equivalent circular diameter. Figure 14 compares the pole figures for the four samples, with an example of the IPF maps provided in Figure 15. Figure 16 and Figure 17 show a comparison between the band contrast maps and kernel misorientation maps, respectively, for two of the four samples. Both the Kearns values and the pole plots indicated these tubes essentially exhibit the same microstructure, with no discernible impact of the combined pre-treatment and activation treatment. Grain size is slightly higher in a-1-05, possibly because of the lower indexing rate due to a poor polish, which may have missed smaller grains and pushed the size to a larger average size. Since we did not observe a difference in microstructure between non-post heat-treated and post-heat-treated specimens, we did not perform getter rate tests on a-specimens.

## 3. Discussion

In this study, we obtained further insight into correlating hydrogen getter surface composition and texture with heat treatment procedures and hydrogen absorption rates. Surface finish and composition is important when the material coating will either be used to facilitate hydrogen absorption or to prevent hydrogen ingress depending on the application of the material.

This study revealed the role of two post-plating heat treatments of nickel-plated zircaloy-4 getters (NPGs) on hydrogen gettering rates. The nickel plating was applied to prevent oxidation of Zircaloy-4 surface and also acted as a catalyst to enhance gettering. Typically, the nickel-plating must be heat treated before desirable gettering can occur. Our NPG getter with historically known satisfying performance was heat treated in air followed by activation heat treatment in a vacuum at a higher temperature. Here, we were interested in finding out if both heat treatment steps were necessary to obtain a desirable gettering performance or if one step could be omitted.

We showed that getter rate performance is indeed dependent on specific heat treatment steps of the nickel surface. The specimens that had only been activated had slower gettering rates than the specimens that had been both heat-treated in air followed by activation. We clarified that the heat treatments did not have an impact on the level of cold work by analyzing the texture of specimens that had no post-plating heat treatment with specimens that had been going through both heat treatment steps. XPS analysis of surface composition showed that the first heat treatment step removed carbon species, which could be correlated with an improved getter rate. The carbon species appeared to be introduced during the nickel electroplating procedure. Therefore, we can conclude that both heat treatment steps are necessary in order to obtain desirable getter rates.

When developing new materials to be used either for hydrogen storage or for hydrogen barriers, it is important to correlate surface composition with hydrogen uptake performance. Future in-depth studies of a catalytic metal coating on a hydrogen absorbing substrate are needed to correlate hydrogen gettering performance with surface characteristics, including evaluating coating thickness, coating coverage, composition, intermetallic phases, microstructure, texture, composition, etc.

## 4. Materials and Methods

### 4.1. Getter Specimens

The Zircaloy-4 getter tubes were fabricated to ASTM B-353 standards by Veridiam Inc. (El Cajon, CA, USA). The nickel-plating was applied by Hohman Plating LLC (Dayton, OH, USA) on both outer (OD) and inner (ID) diameters of the tube per an electroplating procedure and were 0.3–0.5 mils (1 mils = 0.001″; 0.13 mm) thick. Nickel-plated getter (NPG) tube specimens of 2-inch length were cut from longer tubes of OD of 8.25 mm (0.325 inch) and ID of 7.87 mm (0.310 inch).

In order to obtain desirable gettering rates, a getter tube that had been plated with nickel was exposed to two heat treatment steps, referred to as pretreatment and activation. Pretreatment was performed at 425 °C in air for two hours. Activation heat treatment was thereafter performed at 600 °C in a vacuum for one hour to bond nickel to the zirconium-4 substrate [31]. The getter surface remained oxidized (NiO), as could be observed by the blue/purple color. Four different getter tubes were prepared under different heat-treatment conditions. We had a total of eight longer tubes; two of each with the heat treatment condition. Testing was performed to evaluate effects of post-plating treatments, to correlate the effects of those treatments to gettering rates, surface composition and on textures in the Zircaloy.

(a) NPGs with neither pretreatment nor activation (Tubes 1 and 2)
(b) NPGs with pretreatment but without activation (Tubes 3 and 4)
(c) NPGs with activation but without pretreatment (Tubes 5 and 6)
(d) NPGs with pretreatment in air and activation heat treatment (Tubes 7 and 8)

To better understand the role of the post-plating pretreatment step and whether it could be excluded in order to reduce fabrication cost, we measured getter rates of specimens that had not been pretreated, but activated and compared measured getter rates with a getter that had been both pretreated and activated. The tests reported revealed the impact of excluding pretreatment before activation.

### 4.2. Hydrogen Getter Rate Test Equipment

To measure gettering rates, we used a custom-built vacuum system equipped with 10 Torr Baratron to measure pressures down to 0.001 Torr. The nickel-plated Zircaloy-4 specimen (NPG) was placed in a quartz tube that was enclosed in a Blue Lindberg furnace. The quartz tube was connected to stainless steel tubing using a glass-metal transition to seal it and evacuated to a high vacuum before a leak test was performed. Hydrogen gas was then inserted into the system by using a calibrated capillary leak on a H_2_ gas bottle (99.999%, ultra-high purity) with a leak-rate of 2.6·10^−8^ mol/s (calibrated by Vacuum Technology Inc., Oak Ridge, TN, USA). The leak rate of the hydrogen bottle was calculated from a calibration curve to be about 5.7·10^−5^ atm-cc/s depending on the bottle pressure as read on the gauge on the bottle. A thermocouple on the outside of the quartz tube recorded the temperature. The getter rate tests were performed at 375 °C ± 2 °C. After heating to the test temperature, the H_2_ gas was inserted into the quartz tube and pressure was monitored with a capacitance manometer (<10 Torr). When hydrogen comes in contact with the oxidized getter surface, the NiO is reduced to Ni within a few minutes, which is obvious from observing the color changing from blue to metallic.

The gettering rate procedure was performed by first allowing hydrogen absorption to reach a plateau pressure when the absorption rate was the same as the bottle leak rate, and then closing the valve to allow the getter to absorb the remaining gas. Gettering rates were measured for initial hydrogen pick-up in the solid solution α-phase region (H/Zr < 0.02). The gettering rate reported here was assessed from the slope when hydrogen pressure decreased from plateau pressure upon absorption during 60 s. Although we did not perform a full kinetics analysis, it provides a comparison between the specimens, which is what we were interested in to be able to correlate getter rates with surface composition and texture after heat treatment.

### 4.3. X-ray Photoelectron Spectroscopy

Selected areas on each of the samples were examined with X-ray photoelectron spectroscopy (XPS) to characterize the chemical composition of atoms on the surface of nickel-plated getter tube samples. The intent of these examinations was to examine the surfaces for evidence of substances that may be detrimental to the dissociation and/or diffusion of hydrogen, and whether these substances are modified or removed by the pretreatment and/or thermal activation process.

XPS measurements were performed with a Physical Electronics Quantera Scanning X-ray Microprobe. This system uses a focused monochromatic Al Kα X-ray (1486.7 eV) source for excitation and a spherical section analyzer. The instrument has a 32-element multichannel detection system. The X-ray beam is incident normal to the sample and the photoelectron detector is at 45° off-normal. XPS depth profiles were performed using 2 kV monoatomic Ar+ ions rastered over a 3 mm^2^ area of the sample surface and an ion incident angle of 45°. High energy resolution spectra were collected using a pass-energy of 69.0 eV with a step size of 0.125 eV. XPS combined with monoatomic Ar+ ion depth profiling provides valuable chemical state and compositional information of various elements present at surface and subsurface regions. XPS depth profiling was performed using a focused beam of 2 kV, Ar+ ions rastered over a 3 mm^2^ area of the sample using an incident angle of 45°.

### 4.4. Scanning Electron Microscopy

Texture analysis was performed using electron backscatter diffraction (EBSD) to assess the degree of cold work and to learn differences between post-plated heat treatment compared with no post-plating heat treatment. Each thinned walled tube was carefully sectioned down the center of the tube, then one half piece was mounted to ensure the planar surface of the tube cross-section was flush with the mount surface to avoid any tilt effects on the pole figures and orientation maps. The samples were ground through successive SiC grits, polished to a 1 µm diamond and then given a final colloidal silica polish, which yielded a sample that polarized in the optical microscope. It was found that the thinned walled tubes had an issue with beveling on the outside and inside edges due to preferential polishing at the Ni coating, which did not allow for texture measurements on the edges of the tube due to improper polishing and also excess charging from the nearby epoxy. The charging was overcome to a large extent by sputtering a 6 nm coating of carbon, but the EBSD maps were still taken from the center region away from the epoxy as the charging persisted, distorting the beam and pattern. This was consistently done across all of the samples, providing a comparative texture from the center region of each of the measured tubes.

The EBSD maps were taken on a JEOL 7600F FEG-SEM equipped with an Oxford Instruments AZtec system using a Symmetry CMOS detector (MA, USA). The data was acquired with the AZtec Nanoanalysis software package and the data was analyzed using the AZtec Crystal package. The EBSD maps were taken using a 20 keV current and a probe current setting of 16, with a working distance of 24 mm at a tilt of 70 °C. The zirconium was indexed from the NIST structural database in AZtec Crystal using a hexagonal crystal system; Laue group 9 (6/*mmm*), Space Group 194 (P6_3/*mmc*), lattice parameters were a = b = 3.23 Å, c = 5.1477, α = β = 90°, γ = 120°.

**Figure 18:**
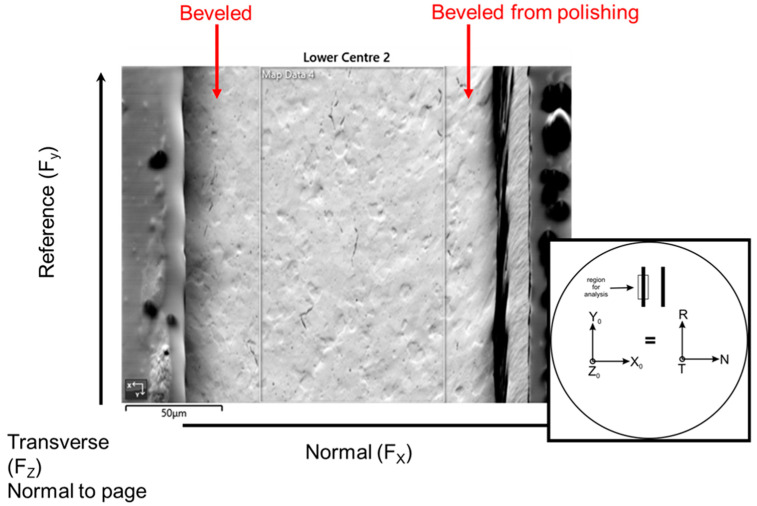
A secondary electron image is shown with a schematic showing the orientation of the sample along with the nomenclature for the orientations. The black outline highlights the center portion of the thin walled tube from which the EBSD data was taken. The colloidal silica preferentially attacked the outer regions, where the Ni plating resides, and created a beveled surface.

A map was obtained over a region approximately 100 × 175 µm in width at 0.25 µm steps. For most of the samples, the hit rate was 80% or greater, but one sample only achieved a hit rate of 67%. Smoothing of the patterns was limited to no more than a 10% improvement. The orientation of the samples is shown in Figure 18; this nomenclature was used throughout the analysis. Orientation maps, pole figures and kernel average misorientation maps were generated from each dataset to allow a comparison of the texture. Kearn’s values were reported for each dataset. No attempt was made to analyze for other phases, such as hydrides or second phase particles.

## 5. Conclusions

We correlated hydrogen getter rates with surface composition and texture after post-plating heat treatment. The getter rate tests of the 16 nickel-plated ziraloy-4 getter specimens show that the samples that were only activated without pretreatment (c-samples) do not initially pick up hydrogen as well as the samples that were pretreated before activation (d-samples) and, therefore, the plateau pressures of the c-samples are in the range of 2–6 times higher than the d-samples, with 5–7 times slower gettering rates. XPS analysis showed that samples without pretreatment (a-samples) consist of carbon, oxygen and nickel, and that pretreatment (b-samples) removed the surface carbon from the nickel surface. XPS also revealed that oxygen diffuses into the nickel during the activation process.

- The surface of a nickel-plated getter tube predominantly consisted of carbon, oxygen and nickel. Through several narrow scan analyses, the layer averaged approximately 55% C, 30% O and 13% Ni.
- The pretreatment process essentially removed all of the surface carbon from the nickel surface that stemmed from the electroplating procedure. The surface was found to consist of nickel and nickel oxide. The depth of nickel oxide on the surface was roughly 2 nm.
- The surface of the pretreated and activated specimen consisted of oxygen and nickel. The depth profile of oxygen was more gradual than that observed in the as-plated material, indicating that surface oxygen diffuses into the nickel during the activation process. It is not known what fraction of oxygen is removed from the surface during activation, and what fraction diffuses into the nickel.

The EBSD texture analysis did not find a difference in texture between non-pretreated, non-activated and getter specimens with both heat treatment steps. Hence, there was no impact on the percentage of cold work during heat treatment. The gettering rate did not appear to be due to the texture since the texture appeared to be the same, but a non-pretreated getter had significantly lower gettering rates, likely due to a poisoned surface from the electroplating procedure; thus, the difference in gettering was related to surface contaminations rather than texture.

## Figures and Tables

**Table 1:** Test Matrix for Getter Rate Tests.

Treatment	Tube	Description of NPG Samples’ Nickel Surface Heat Treatment	Number of Getter Rate Samples per Tube
c	5	Only final activation treatment (no pretreatment)	4
c	6	Only final activation treatment (no pretreatment)	4
d	7	Pretreated in air and activation treatment	4
d	8	Pretreated in air and activation treatment	4

**Table 2:** Summary of steady state hydrogen plateau pressure and calculated getter absorption rate constants.

Specimen ID	Plateau Pressure (Torr)	Calculated Rate Constants (s^−1^)
c-5-01	0.638	2.84∙10^−3^
c-5-02	0.987	1.79∙10^−3^
c-5-03	0.761	2.35∙10^−3^
c-5-04	0.579	2.70∙10^−3^
c-6-01	1.379	1.31∙10^−3^
c-6-02	0.798	2.24∙10^−3^
c-6-03	0.615	2.89∙10^−3^
c-6-04	0.506	3.49∙10^−3^
d-7-01	0.222	7.49∙10^−3^
d-7-02	0.318	5.93∙10^−3^
d-7-03	0.314	5.99∙10^−3^
d-7-04	0.241	5.81∙10^−3^
d-8-01	0.219	8.79∙10^−3^
d-8-02	0.316	5.59∙10^−3^
d-8-03	0.245	8.79∙10^−3^
d-8-04	0.249	7.34∙10^−3^

**Table 3:** Test Matrix for XPS analysis of NPG specimens.

Treatment	Description of NPG Samples’ Nickel Surface Heat Treatment	Number of NPG Samples per XPS Analysis
a	No heat treatment	1
b	Only pretreatment (no activation)	1
d	Pretreated in air and activation treatment	1

**Table 4:** Test Matrix for EBSD Texture Analysis.

Treatment	Tube	Description of NPG Samples	Number of Samples for EBSD per Tube
a	1	Untreated	1
a	2	Untreated	1
d	7	Pretreated in air and activation treatment	1
d	8	Pretreated in air and activation treatment	1

**Table 5:** Summary of measured Kearn’s values for the four NPG samples.

Sample ID	Kearn’s Values			Number of Grains Equivalent Circle Diameter (µm)	
	fn	ft	fr		
a-1-05	0.653	0.296	0.051	913	9
a-2-05	0.665	0.283	0.052	1186	6.7
d-7-05	0.675	0.271	0.053	1227	6.76
d-8-05	0.682	0.273	0.045	1129	7.1

## Data Availability

Not applicable.
